# Single cell transcriptomes reveal expression patterns of chemoreceptor genes in olfactory sensory neurons of the Caribbean spiny lobster, *Panulirus argus*

**DOI:** 10.1186/s12864-020-07034-7

**Published:** 2020-09-22

**Authors:** Mihika T. Kozma, Hanh Ngo-Vu, Matthew T. Rump, Yuriy V. Bobkov, Barry W. Ache, Charles D. Derby

**Affiliations:** 1 Neuroscience Institute, Georgia State University, Atlanta, GA 30303 USA; 2 Whitney Laboratory, University of Florida, St. Augustine, Florida, 32084 USA

**Keywords:** Crustacea, G-protein coupled receptor, Ionotropic receptor, Olfaction, Olfactory sensory neuron, Single cell transcriptome, Spiny lobster, TRP channel

## Abstract

**Background:**

Crustaceans express several classes of receptor genes in their antennules, which house olfactory sensory neurons (OSNs) and non-olfactory chemosensory neurons. Transcriptomics studies reveal that candidate chemoreceptor proteins include variant Ionotropic Receptors (IRs) including both co-receptor IRs and tuning IRs, Transient Receptor Potential (TRP) channels, Gustatory Receptors, epithelial sodium channels, and class A G-protein coupled receptors (GPCRs). The Caribbean spiny lobster, *Panulirus argus*, expresses in its antennules nearly 600 IRs, 17 TRP channels, 1 Gustatory Receptor, 7 epithelial sodium channels, 81 GPCRs, 6 G proteins, and dozens of enzymes in signaling pathways. However, the specific combinatorial expression patterns of these proteins in single sensory neurons are not known for any crustacean, limiting our understanding of how their chemosensory systems encode chemical quality.

**Results:**

The goal of this study was to use transcriptomics to describe expression patterns of chemoreceptor genes in OSNs of *P. argus*. We generated and analyzed transcriptomes from 7 single OSNs, some of which were shown to respond to a food odor, as well as an additional 7 multicell transcriptomes from preparations containing few (2–4), several (ca. 15), or many (ca. 400) OSNs. We found that each OSN expressed the same 2 co-receptor IRs (IR25a, IR93a) but not the other 2 antennular coIRs (IR8a, IR76b), 9–53 tuning IRs but only one to a few in high abundance, the same 5 TRP channels plus up to 5 additional TRPs, 12–17 GPCRs including the same 5 expressed in every single cell transcriptome, the same 3 G proteins plus others, many enzymes in the signaling pathways, but no Gustatory Receptors or epithelial sodium channels. The greatest difference in receptor expression among the OSNs was the identity of the tuning IRs.

**Conclusions:**

Our results provide an initial view of the combinatorial expression patterns of receptor molecules in single OSNs in one species of decapod crustacean, including receptors directly involved in olfactory transduction and others likely involved in modulation. Our results also suggest differences in receptor expression in OSNs vs. other chemosensory neurons.

## Background

**Fig. 1:**
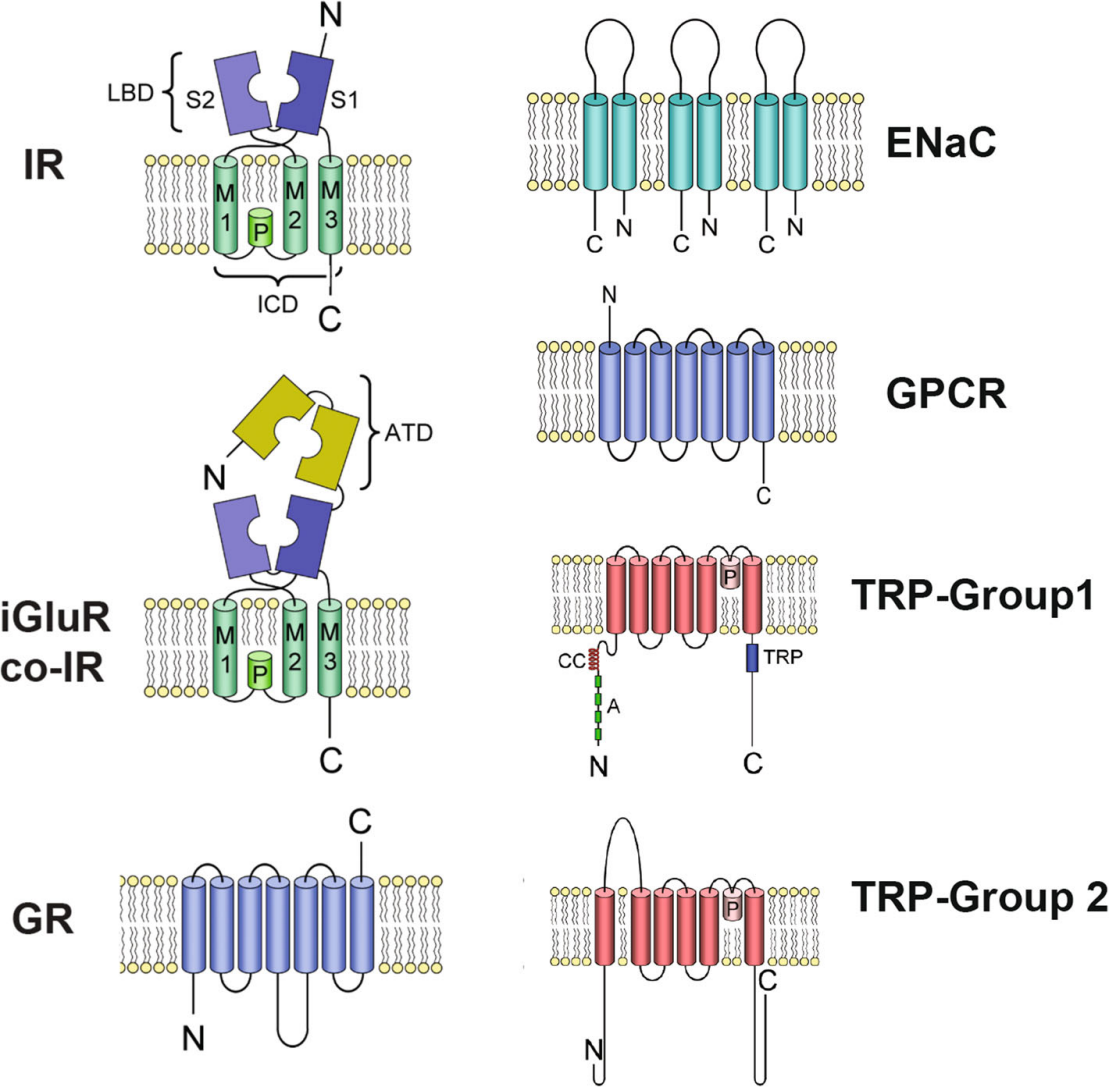
Schematic drawing of the molecular structure of putative crustacean chemoreceptor proteins. Ionotropic Receptor (IR): co-receptor IR (co-IR), ionotropic glutamate receptor (iGluR), and tuning IR. Gustatory Receptor (GR). Epithelial sodium channel (ENaC). G Protein Coupled Receptor (GPCR). TRP channels (TRP). For coIRs and IRs: transmembrane domains (M1, M2, M3), pore loop (P), ligand binding domain (LBD) S1 and S2, amino terminal domain (ATD), intracellular domain (ICD). For TRP channels: coiled-coil domain (CC), ankyrin repeats (A), TRP domain (TRP)

**Fig. 2:**
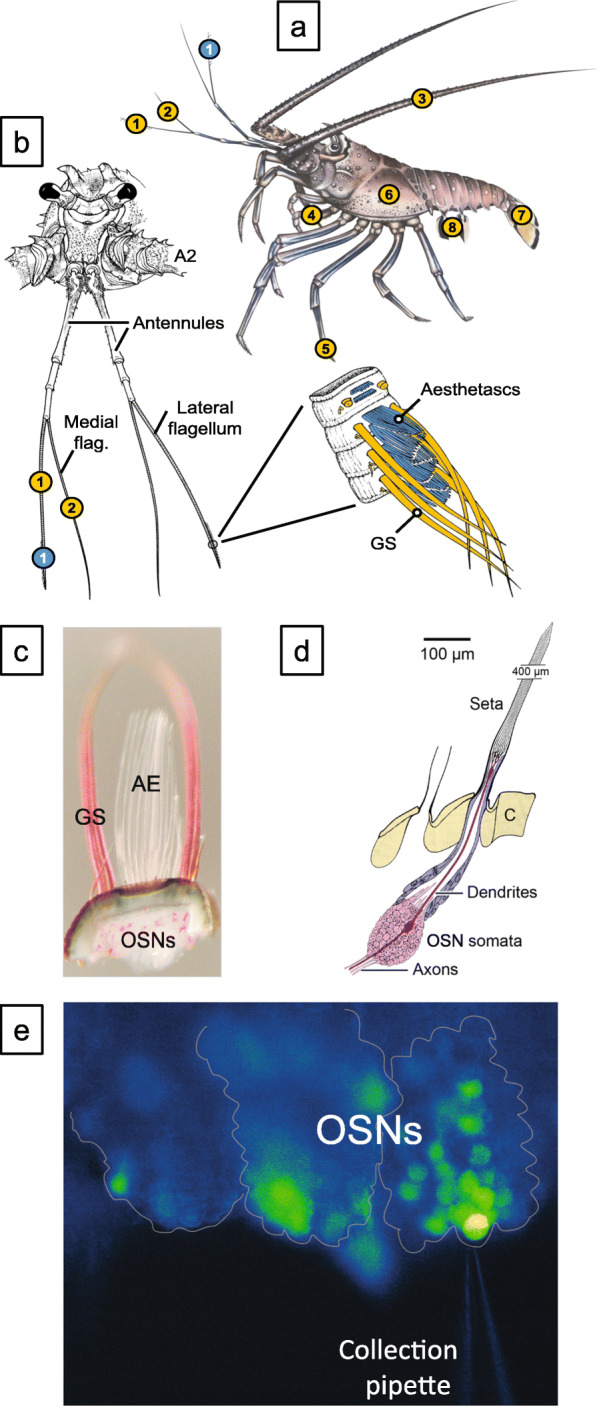
Olfactory organ of the Caribbean spiny lobster *Panulirus argus*, and calcium imaging of odorant-activated responses from olfactory sensory neurons. **a.** Location of aesthetasc sensilla mediating olfaction (blue) and bimodal chemo-mechanosensory sensilla mediating distributed chemoreception (yellow) on different body parts and appendages (1 - lateral flagellum of antennule, 2 - medial flagellum of antennule, 3 - second antenna, 4 - mouthparts, 5 - walking legs, 6 - gill chamber, 7 - tail fan, 8 - pleopods). **b.** Location of aesthetasc sensilla and bimodal chemo-mechanosensory sensilla on the antennules. Aesthetascs (blue) are restricted to a tuft of sensilla on the distal one-third of the lateral flagellum. Bimodal chemo-mechanosensory sensilla (yellow) among them guard sensilla (GS) are associated with the aesthetascs but also occur on the proximal part of the lateral flagellum and on the entire medial flagellum.  **a.** and **b.** are modified from [2]. **c.** Transmitted light microscopic image of antennule slice preparation used for calcium imaging of odorant-evoked responses. The preparation consists of an annulus bearing aesthetasc sensilla (AE), and their olfactory sensory neurons (OSNs), and guard sensilla (GS). **d.** Schematic drawing of a single aesthetasc of *P. argus* (modified from [5]). The aesthetasc is innervated by ~ 300 bipolar olfactory sensory neurons (OSNs) whose somata form a cluster below the cuticle (C). Each soma gives rise to 1) a dendrite projecting into the cuticular seta where it branches into many ciliated outer segments and 2) an axon projecting to the brain. **e.** Image showing the method of collecting odorant-activated OSNs using the calcium imaging preparation. An antennule slice (shown in panel **c**) was prepared for calcium imaging (described in Methods). The dye-filled OSNs, situated in cell clusters that are outlined, are shown with an electrode positioned near a target OSN that responded to an 800-msec pulse of the food odorant 500 mg/l TetraMarine. A video showing odorant-activated responses of OSNs in this preparation is shown in Additional file 1


Crustaceans have well-developed chemical senses because environmental chemicals are key in informing crustaceans about resources [1–4]. Fundamental to understanding how sensory neurons detect chemical information is identifying receptors and other transduction molecules expressed in them. Crustaceans have at least five classes of chemoreceptor proteins. These are variant Ionotropic Receptors (IRs), Transient Receptor Potential (TRP) channels, Gustatory Receptors (GRs), epithelial sodium channels (ENaCs), and class A G protein coupled receptors (GPCRs). The molecular identity (Fig. 1) and sensory organs in which they are expressed (Fig. 2) have been described for four decapod crustacean species, including the Caribbean spiny lobster, *Panulirus argus* [6, 7].

**Additional file 1:** Video of the odorant-activated responses of OSNs. The calcium-imaging preparation shown in Fig. 2e was used to record responses of OSNs to an 800-ms pulse of 500 mg/l TetraMarine. This 52-s recording was accelerated for viewing.

Variant IRs, which are heterotetrameric receptor-channels that evolved from ionotropic glutamate receptors (iGluRs) [8–15], are prevalent in most crustaceans [6, 7, 16–24]. Their function is best studied in insects, especially *Drosophila* and mosquitoes, where they function in chemical sensing [11, 25–34] but also in thermoreception, hygroreception, and circadian rhythms [35–40]. The variant IRs include two structural types (Fig. 1). One type, with two members – IR25a and IR8a – has the full amino-terminal domain (ATD) of iGluRs and a loop (co-receptor extra loop: CREL) in the ligand binding domain (LBD) with a distinct glycosylation site that is critical to their ability to perform their distinctive role of controlling intracellular transport of IRs and their eventual insertion into the dendritic membrane [41]. The other structural type of IR lacks or has truncated ATD, and lacks the distinctive loop (CREL) of the LBD. The heterotetrameric IR is likely composed of two co-receptors (co-IRs), which can include ATD-bearing co-IRs (IR25a, IR8a) or non-ATD-bearing co-IRs (IR93a, IR76b) [41, 42]. The other IRs composing the heterotetramer are “tuning IRs” [41] whose specific combination in the heterotetramer determines the binding specificity of the receptor [25, 41, 43].

Co-IRs and tuning IRs have different evolutionary histories [9, 11, 41, 44]. IR25a is most ancient, being present in protostomes but not in deuterostomes. IR8a, IR93a, and IR76b evolved more recently, in arthropods [9, 23]. Co-IRs mediate unique sensory functions when combined with specific tuning IRs [45, 46]. The tuning IRs IR21a, IR40a, and IR75-family appear to be arthropod-conserved. Several tuning IRs have been identified as insect-conserved or crustacean-conserved IRs, while many other tuning IRs appear to be species specific [6, 7, 9, 23, 41].

Although cellular expression patterns of IRs are well described in the olfactory and gustatory organs of *Drosophila* and other insects [25], the little information on crustaceans is mostly limited to co-IRs. Crustaceans have chemosensory neurons covering their body surface, constituting two major systems that differ in peripheral and central organization: olfaction and distributed chemoreception [2, 3]. Olfaction is mediated by aesthetasc sensilla on the distal end of the lateral flagella (LF) of the first antennae (antennules). Aesthetascs are innervated only, and densely, by olfactory sensory neurons (OSNs) and are thus unimodal sensilla. Distributed chemoreception includes gustation plus other chemical senses except for olfaction. Axons from OSNs project to the olfactory lobe in the central nervous system. Sensilla composing distributed chemoreception are found covering the body and its appendages, including the antennules, second antennae, mouthparts, and legs (Fig. 2). Distributed chemosensilla are structurally diverse but share two features: they are innervated by both chemosensory neurons (CSNs) and mechanosensory neurons (MSNs) and are thus bimodal, and their axons project to regions of the central nervous system different than OSNs [2, 3]. IRs are probably expressed in all chemosensory organs of crustaceans. In most species studied, co-IRs IR25a, IR8a, and IR93a have been shown to be strongly expressed in the LF [6, 7, 16, 21, 24, 47–49] and more weakly expressed in other chemosensory organs [6, 7, 16, 24]. The cellular expression patterns of tuning IRs are more poorly described [6, 7]. Evidence for a functional role of IR25a in chemoreceptor in crustaceans comes from parasitic sea lice, in which RNA interference of IR25a changed their behavioral responses to host chemicals [19, 50].

TRP channels are homo- or heterotetramers consisting of six transmembrane segments (Fig. 1) and are categorized into eight subfamilies (TRPC, TRPA, TRPN, TRPV, TRPM, TRPML, TRPP, TRPS) [51–57], seven of which have been found in crustaceans [6, 7]. TRP channels can detect many types of environmental stimuli, including those involved in olfaction, gustation, photoreception, thermosensation, mechanosensation, and hearing, in some cases acting as downstream integrators of signals from the primary detectors of sensory stimuli [58–60]. Members of TRPA, TRPA1, Painless, TRPL, and TRPC are chemical sensors in olfaction and gustation in insects [51, 52, 61–65], though TRPA1 and Painless also play roles in thermoreception [66]. Homologues of TRPA1, Painless, TRPL, and TRPC occur in crustacean chemosensory organs [6, 7], though a role in chemoreception has not been demonstrated in crustaceans.

GRs are seven-transmembrane ionotropic chemoreceptors that are an ancient lineage [12, 67–69] (Fig. 1). They have expanded families in several groups, particularly insects. GRs are prevalent in some crustaceans – the amphipod *Hyalella azteca* (155 GRs), the branchiopod *Daphnia pulex* (59 GRs), and the copepod *Eurytempora affinis* has (67 GRs) [23, 68, 70, 71] – though it is not known in which tissues these GRs are expressed. Other crustaceans, including decapods, have only one to a few GRs [6, 7, 16, 21, 23, 72].

Epithelial sodium channels (ENaC) are ionotropic receptors (Fig. 1) that *Drosophila* uses to detect salt, water, and pheromones, as well as acting as downstream amplifiers of responses generated by other receptors [73–78]. Crustaceans have ENaCs in their LF and dactyl, but these do not include homologues of the chemosensory *pickpocket* genes ppk23 and ppk28 in insects [7].

Two other types of receptor proteins – G-protein coupled receptors (GPCRs) and ionotropic glutamate receptors, especially NMDA receptors (Fig. 1) – are found in crustacean chemosensory organs [6, 7] (Rump, Kozma, and Derby unpublished data). Class A (rhodopsin-like) GPCRs are major sensory transduction receptors in the vertebrates, including in photoreception, olfaction, and gustation, and even in some invertebrates, they have been shown to play roles in chemical sensing [79–90]. G protein signalling cascades are implicated in crustacean olfaction, and some of the molecules involved have been cloned and sequenced [16, 91–93], but the specific GPCR molecules in this pathway remain unidentified. Some of the crustacean GPCRs may be involved in modulation of chemosensory responses generated through odorant activation of other receptor types, rather than being directly involved in gating odorant responses [6, 7, 16, 22, 42, 52, 94, 95].

To understand mechanisms of neural coding of chemical stimuli by chemosensory systems, it is necessary to know the cellular expression patterns of receptor molecules in individual chemosensory neurons. Given that each crustacean species potentially has dozens to hundreds of different types of chemoreceptor proteins, standard immunocytochemical or in situ hybridization techniques will yield limited information. However, single cell transcriptomic analysis provides a tool to examine expression patterns in individual olfactory and other chemosensory cells [96–108]. Thus, the goal of this study is to use single cell transcriptomics to provide a preliminary view of the combinatorial patterns of chemoreceptor gene expression in OSNs of the Caribbean spiny lobster *Panulirus argus*, a major crustacean model of chemoreception for which chemosensory organ-level transcriptomes are available to be used as a reference database.

## Results

We analyzed 14 transcriptomes that passed quality control criteria for RNA sequencing. Seven of these transcriptomes were from single OSNs (single cells), which we label as single cell transcriptomes SCT1a to SCT1g. The other seven were from preparations containing a few (2, 3, or 4) to many (~ 15 or 400) OSNs, which we label as multicell transcriptomes MCT2, MCT3, MCT4, MCT15a, MCT15b, MCT15c, and MCT400 with the number indicating the number of OSNs in that MCT. MCT400 may have a few other cell types. Table 1 presents features of these transcriptomes, including number of OSNs, evidence of responsiveness of OSNs to chemical stimulation, and whether they were bursting OSNs [109–111]. Raw reads from SCTs and MCTs were mapped to our previously generated *P. argus* reference transcriptome [7] using RSEM [112]. We analyzed for expression of receptor genes that were previously characterized in tissues of *P. argus*: antennular lateral flagellum (LF), dactyl of the leg (dactyl), and brain [7] (Rump, Kozma, and Derby unpublished data) (Fig. 2), including variant IRs, TRP channels, GRs, ENaCs, NMDArs, and class A GPCRs. We also examined expression of G proteins and other molecules in the signaling pathways.

**Table 1:** Summary of features of the 14 transcriptomes in this study. SCT = Single cell transcriptome. MCT, multicell transcriptome. OSN, Olfactory Sensory Neuron. QC, Quality Control. TET, TetraMarine™, a food stimulus

Prep	# of OSNs	QC (RNA ng/μL)	Response Characteristics
SCT1a	1	0.262	Not tested for chemosensitivity.
SCT1b	1	0.390	Not tested for chemosensitivity.
SCT1c	1	0.292	Responsive to TET. Spontaneous bursting cell.
SCT1d	1	0.410	Responsive to TET. Spontaneously bursting cell.
SCT1e	1	0.502	Responsive to TET.
SCT1f	1	0.776	Responsive to TET.
SCT1g	1	0.376	Responsive to TET and Glutamate.
MCT2	2	0.588	Not tested for chemosensitivity.
MCT3	3	0.488	Not tested for chemosensitivity.
MCT4	4	0.524	Not tested for chemosensitivity.
MCT15a	~ 15	1.408	Not tested for chemosensitivity.
MCT15b	~ 15	4.180	Some cells in cluster are responsive to TET.
MCT15c	~ 15	4.340	Some cells in cluster are responsive to TET.
MCT400	~ 400	7.380	Not tested for chemosensitivity.

### Variant IRs

The extracellular ligand binding domain (LBD) of variant IRs consists of two half-domains (S1, S2). A total of 582 variant IRs from *P. argus* were used in these analyses, including sequences having either S1, S2, or both binding domains [7]. We used all of these sequences, for two reasons. First, we constructed maximum likelihood phylogenetic trees with the sequences and found that the three groups of sequences clustered with each other and with IRs from other species, supporting the view that all are IRs. Second, we found that some of the S1 only and S2 only sequences were the most abundant sequences in the SCTs and MCTs, supporting the view that they are IRs and thus making them essential for understanding the receptor expression patterns in these transcriptomes.

Variant IRs are the most highly expressed receptor genes in SCTs and MCTs. Based on transcripts per million (TPM) that were estimated using RSEM, each SCT had 3–4 IRs that are amongst the top 20 most highly expressed genes in the transcriptomes. Similarly, MCTs had 2–5 IRs within the top 50 most highly expressed genes. Every SCT and MCT expressed two of the four co-IRs present in the LF transcriptome: IR25a and IR93a (Additional file 2, Fig. 3). These were expressed in high abundance and were one of the 20 or 30 most abundant sequences in the transcriptomes. IR25a had 8549 TPM per SCT (median, range of 6665 to 12,281) and IR93a had 15,707 (median, range of 8455 to 23,795) (Additional file 2, Fig. 3). The ratio of the total TPMs for IR25a vs. IR93a in each OSN was 0.53 (median, range of 0.43 to 1.11). On the other hand, none of the SCTs or MCT expressed the other two co-IRs that were detected in the LF tissue, IR8a or IR76b, with one exception. This was MCT400, which had very low expression of IR8a, and this transcriptome likely contains other cell types besides the 400 OSNs such as tegumental gland cells and auxiliary cells.

**Fig. 3:**
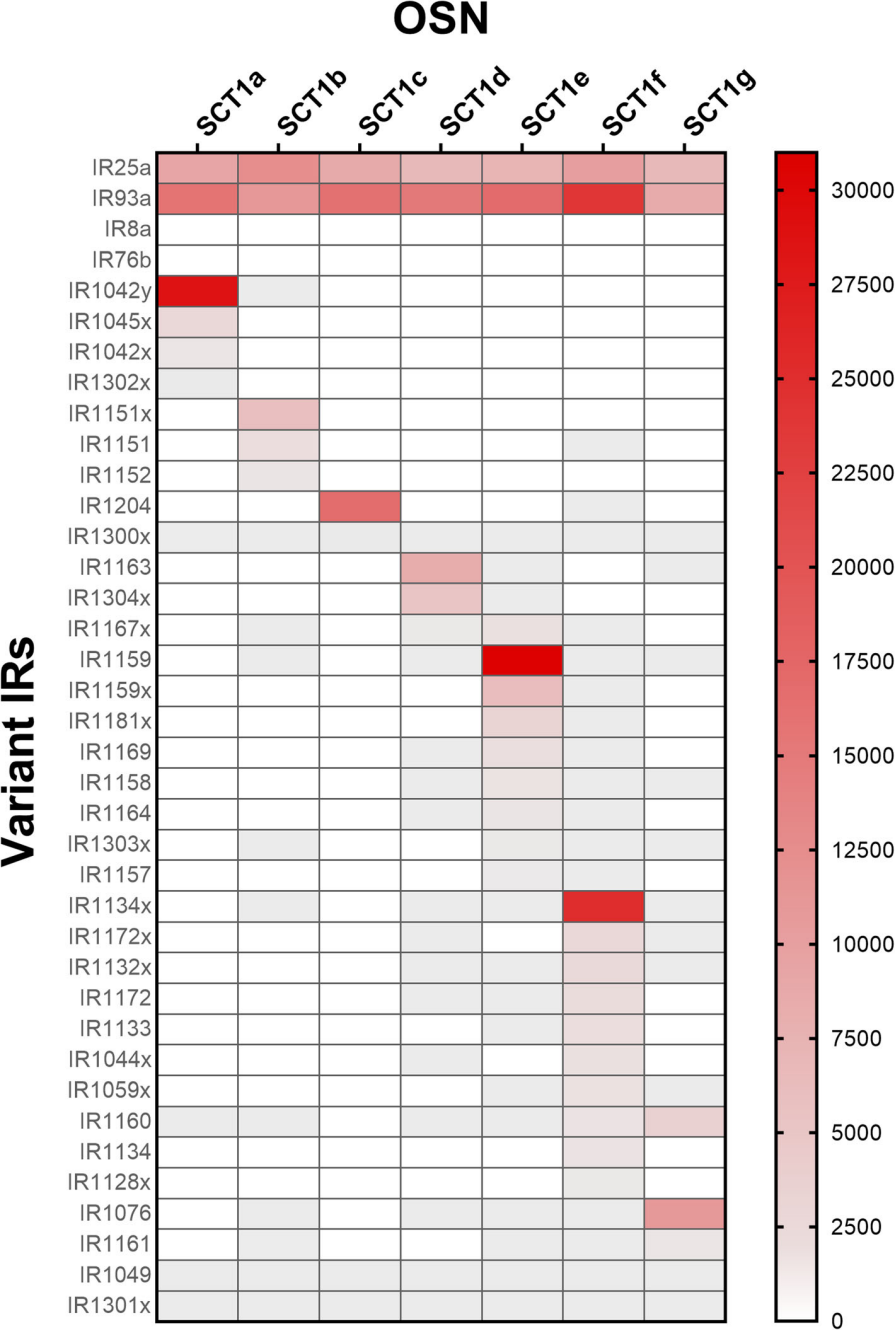
Variant IR gene expression in seven single OSNs. Shown is the TPM of the most highly expressed variant IRs genes for each of the seven single OSNs in a double gradient heatmap. OSNs are SCT1a to g on the x-axis, sequences IDs are on the y-axis. Smallest value set at 0 TPM indicated by white, baseline value set at 0.02 TPM indicated by grey, and largest value set at 31,000 TPM indicated by red. Shaded regions indicate TPM value corresponding to the color gradient shown in the scale bar

Each SCT expressed many tuning IRs but only a few had high expression (Additional file 2, Fig. 3). The median number of tuning IRs with one or more TPM per SCT was 29 (range of 9 to 53) of the 578 tuning IRs. Collectively, the seven SCTs expressed 104 of the 578 tuning IRs. The most abundant tuning IR in each SCT had 16,785 TPM (median, range of 5879 to 30,812), representing 66.7% (median, range of 59.4 to 99.1%) of the total TPM of tuning IRs in the cell. Two of the seven SCTs had a single dominant tuning IR that represented > 88% of the total TPM of tuning IRs in that SCT. For five of the SCTs, the most abundant one to three tuning IRs accounted for > 95% of the total TPM of IRs in that SCT, and for the other two SCTs, six to eight tuning IRs were required to account for > 95% of the total TPM of IRs. The dominant tuning IRs in the SCTs were IR1042y, IR1134x, IR1151x, IR1159, IR1163, IR1304x, and IR1204. Thus, although SCTs express many types of IRs, one to eight IRs accounted for most of the transcripts.

The MCTs contained a more diverse set of tuning IRs than the SCTs (Additional file 2). The median number of tuning IRs with one or more transcripts per MCT was 54 (range of 23 to 219). Furthermore, the tuning IRs expressed in the MCTs differed from each other. As expected, the MCTs containing more OSNs (MCT15a, MCT15b, MCT15c, MCT400) expressed the greatest number of different tuning IRs, and the most highly expressed tuning IRs accounted for < 10% of the total number of tuning IR transcripts in the MCTs. Collectively, the seven MCTs expressed 345 of the 578 tuning IRs, and the SCTs and MCTs collectively expressed 361 of the 578 tuning IRs.

**Fig. 4:**
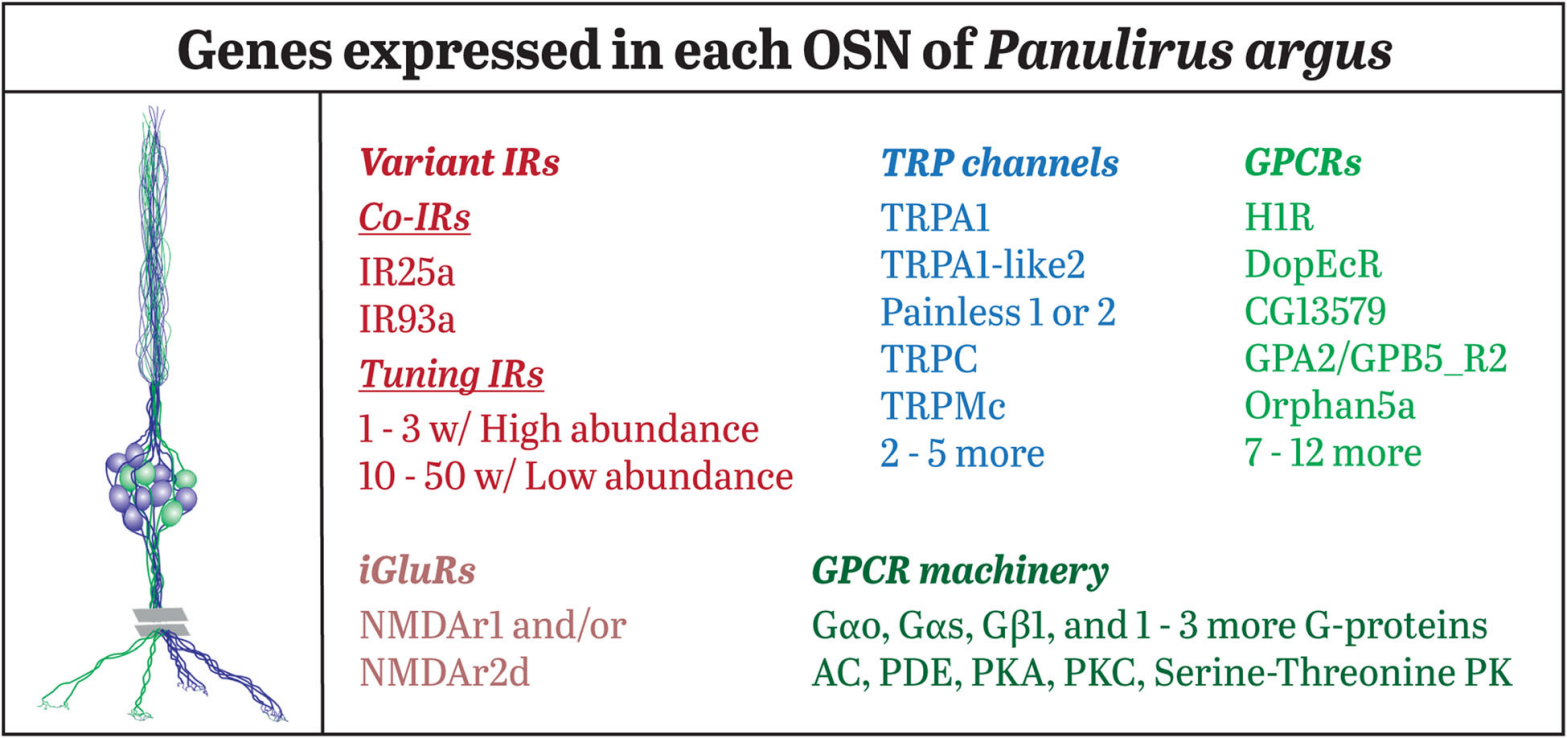
Summary of gene expression in single olfactory sensory neurons of the Caribbean spiny lobster, *Panulirus argus.* Each of the seven single cell transcriptomes expresses all of the mentioned molecules, plus an additional number of genes belonging to each class of molecules

Several other features of these transcriptomes are noteworthy. First, the ratio of the TPM for co-IRs vs. tuning IRs was 1:1 for both the SCTs (median 1.0, range of 0.5 to 2.6) and the MCTs (median 1.0, range of 0.5 to 1.5). Second, only three tuning IRs – IR1049, IR1301x, and IR1300x – were expressed in every SCT and MCT, and their expression was at relatively low levels (< 160 TPM). Third, tuning IRs expressed in the SCTs and MCTs were much more likely to have higher expression levels in the *P. argus* LF transcriptome than the dactyl or brain transcriptome. In fact, all tuning IRs that had > 100 TPM in an SCT or MCT had higher expression in the LF than the dactyl or brain transcriptomes (DESeq2 analysis from [7] – Fig. 4b and S4 table; Additional files 8 and 9). Fourth, tuning IRs with 15 or more TPM in a SCT or MCT were always more highly expressed in the LF transcriptome than the dactyl transcriptome (DESeq2 analysis from [7] – Fig. 4b and S8 table/Additional file 9). Fifth, the IRs in *P. argus* that are conserved in different phylogenetic groups, e.g., in arthropods, crustaceans, or decapod crustaceans [7], were present in SCT and MCT, but not abundantly so. Of the arthropod-conserved tuning IRs (IR21a, IR40a-family, IR68a, IR75-family [IR1034, IR1091–1095], IR1039), only three (IR40a-3, IR40a-9, IR1039) were expressed in the transcriptomes (two SCTs and three MCTs). Of the five crustacean-conserved tuning IRs (IR1020, IR1038, IR1064, IR1066, IR1067), only one (IR1038) was expressed and only in one MCT. Of the 12 decapod conserved tuning IRs, only one (IR1155) was expressed and only in one MCT. The remainder of the tuning IRs expressed in SCT and MCT are, to the best of our current knowledge, species-specific IRs.

### TRP channels

The LF transcriptome has 17 types of TRP channels belonging to seven of the eight families of the two groups (Additional file 3). While all TRP channels were detected in both the LF and dactyl tissues, only TRPA5–1 and TRPgamma had higher expression in the LF compared to the dactyl [7]. Of these 17 types, 12 were expressed in at least one of the seven SCTs and 15 were expressed in at least one of the 14 SCT or MCTs. In fact, each of the seven SCTs expressed 5 to 10 of these TRP channel types. Each SCT expressed the following five types: TRPA1, TRPA1-like2, Painless2 or 1, TRPC, and TRPMc (a decapod crustacean specific TRP channel) (sequence IDs from [7]). The levels of expression were moderate: the total number of TRP channel transcripts in SCTs was 69 TPM (median, range of 23 to 163 TPM). Painless2, TRPA1, and TRPMc were the most abundantly expressed, with Painless2 being the dominant TRP channel in four of the seven SCTs and TRPA1 or TRPMc being the most abundant in the other three SCTs. We did not find evidence for a member of the newly recognized family of TRP channels, TRPS [57], in any of our transcriptomes. The MCTs had higher total TPM for TRP channels and more TRP channel types than did the SCTs, where the MCTs with 15 or more cells expressed 10 to 12 TRP channel types. Overall, TRP channels had much lower expression (TPM) compared to IRs in each SCT and MCT.

### GRs and ENaCs

A single GR – GR1 (evg1904265) – was identified in the LF of *P. argus*, and while it had low expression in the LF transcriptome, it was three-fold more highly expressed in the LF than in the dactyl transcriptome and it was not detected the brain transcriptome [7]. GR1 was not present in any SCT and was only in MCT400 and in low abundance (12 TPM). Eight types of ENaCs were present in the LF and dactyl transcriptomes of *P. argus*, with 1 (ENaC4) more highly expressed in LF and 3 of the 8 being more highly expressed in the dactyl (Additional files 4 and 9). Only one of these eight ENaC (ENaC7) was found in the 14 transcriptomes, and that was only in MCT400 and in very low abundance (< 1 TPM).

### NMDArs

NMDA receptors (NMDArs) are another class of ionotropic glutamate receptors (iGluRs) expressed in OSNs of crustaceans [6, 7]. NMDArs were expressed in every SCT, with one or two different types per SCT (Additional file 5). Five types of NMDA receptors were identified in *P. argus* [6, 7], and four of these were expressed in at least one of the 14 SCTs or MCTs (Additional file 5). These were NMDAr1, NMDAr2a, NMDAr2b, and NMDAr2d; NMDAr3 was not expressed in any SCT or MCT. NMDAr2b and NMDAr2d were more highly expressed in the brain than the LF transcriptome (Additional files 5 and 8). Each SCT had NMDAr1 and/or NMDAr2d. NMDAr1 was most prevalent, being expressed in six of the seven SCTs, and NMDAr2d was next most prevalent, in four of the seven SCTs. Two NMDA receptors – NMDA2a and NMDA2b – were found only in the MCTs. The total number of transcripts for all NMDArs combined per SCT was 10.4 TPM (median, range of 3.0 to 27 TPM).

### GPCRs and G proteins

A diverse set of ca. 100 members of class A GPCRs was found in the combined LF-dactyl-brain transcriptome assembly of *P. argus*, 81 of which were expressed in the LF (Rump, Kozma, and Derby, unpublished data; Additional files 6, 10). Of these 81 GPCRs in the LF, 54 were expressed in the 14 transcriptomes of our study, 28 across the seven SCTs, and all 54 across the seven MCTs. These included representative GPCRs for the following: 1) 13 amine receptors: histamine (H1R), serotonin (5HT1A, 5HT2A, 5HT7), dopamine (Dop1⍺R, Dop1βR, Dop2⍺R), octopamine (Octβ2R, Octβ3R, Octβ4R), tyramine (Tyr1), dopamine/ecdysteroid (DopEcR), trace amines (TAAR-like); 2) two acetylcholine receptors (mAChRA, mAChRB); 3) one purinergic receptor (adenosine); 4) 13 neuropeptide receptors: allatostatin C (AstC-R2, AstC-R3), trissin (trissinR), tachykinin (tachykninR99D), natalisin (natalisinR1), FMRFamide (FMRFamideR), inotocin (inotocinR2, inotocinR-like), short neuropeptide F (sNPFR), cardioacceleratory peptide (CCAPR), elevenin (eleveninR1, eleveninR-like, ecdysis triggering hormone (ETHR1b)); 5) five leucine-rich repeat containing GPCRs (bursiconR1, bursiconR2, GRL101, GPA2/GPB5_R1, GPA2/GPB5_R2); 6) one fatty acid receptors (prostaglandinE2R2); 7) two opsins (onychopsin-like, long wave length sensitive (LWS) opsin1); 8) six characterized orphan receptors whose physiological functions are identified but their endogenous ligands are generally not (CG13579, CG13995, GPR84, GPR142, GPR161, HP1R); and 9) 11 uncharacterized orphan receptors (Orphan2, 3, 3x, 4, 5a, 5b, 7, 8, 9a, 9b, 15) (Additional files 6, 10).

Each SCT expressed 12 to 17 different types of GPCRs. The MCTs expressed even more types, as expected, with 16 to 40 per MCT and a positive correlation between the number of OSNs and the number of types of GPCRs in these MCTs. Five GPCRs were expressed in all seven SCTs: histamine receptor (H1R), dopamine/ecdysteroid receptor (DopEcR), CG13579 (an orphan receptor in *Drosophila* with high sequence similarity to the dopamine/ecdysteroid receptor); leucine-rich repeat-containing GPCR (GPA2/GPB5_R2), and an orphan receptor (Orphan5a). Notable for also being relatively highly expressed in the SCTs are other amine receptors: two serotonin receptors (5HT1AR and 5HT7R), one octopamine receptor (Octβ3R), one tyramine receptor (TyrR1), one trace amine-associated receptor-like (TAAR-like), two acetylcholine receptors (mAChRA, mAChRB), two neuropeptide receptors (AstC-R2, TrissinR), an opsin (onychopsin-like), three characterized orphan receptors (CG13995, GPR84, GPR161), and three uncharacterized orphan receptors (Orphan2, Orphan4, Orphan5b). The total number of GPCR TPM per SCT was 19,771 (median, range of 7703 to 26,855). The most abundant single GPCR expressed in the SCTs had 4849 TPM (median, range of 2708 to 6841 TPM), representing 26.8% (median, range of 18.3 to 33.9%) of the total number of GPCR transcripts in that SCT (SCT1e).

Multiple types of G proteins were also found in the SCTs. Four Gα subunits were identified, in the following relative abundances in the SCTs: Gαo > Gαs > Gαq > Gαi (Additional file 7). Gαo and Gαs were found in each of the seven SCTs, while Gαq and Gαi were found in four and one of the SCTs, respectively. Two Gβ subunits (Gβ1, Gβ2) were also found in all or most of the SCTs, with Gβ1 being expressed in much higher abundance than Gβ2. The expression levels of Gα subunits and Gβ subunits were 212 TPM (median, range of 182 to 228) and 234 TPM (median, range of 166 to 448) respectively, with a Gα:Gβ ratio of 0.84 (median, range of 0.51 to 1.29).

### Signaling cascades

Many chemicals in the cAMP, IP3, and other signaling cascades were identified in the SCTs and MCTs (Additional files 7, 10). These include many varieties of adenylate cyclase (AC), phosphodiesterase (PDE), protein kinase A (PKA), phospholipase C (PLC), protein kinase C (PKC), and serine-threonine protein kinase. Most of these were expressed in many and in some cases all of the SCTs and MCTs.

### Summary

From our analysis of transcriptomes of seven SCTs and seven MCTs, we have identified receptor and associated molecules expressed in OSNs of *P. argus*, some expressed in every OSN and others with variable expression across the cells. This expression pattern in single OSNs is represented in Fig. 4. Each OSN expressed the following molecules: 1) Two co-IRs IR25a and IR93a (but not IR8a or IR76b) as well as 29 (median, range 9–53) tuning IRs, although only a few tuning IRs accounted for most of the transcripts per OSN and the expression pattern of tuning IRs was different across the OSNs. 2) Seven TRP channels (median, range of 5 to 10) including the same five (TRPA1, TRPA1-like2, TRPC, TRPMc, and Painless2/Painless1). 3) One or two NMDArs. 4) Nineteen class A GPCRs (median, range of 14 to 42 range), especially receptors for histamine, acetylcholine, serotonin, octopamine, and allatostatin. 5) Several types of G proteins, including Gαo, Gαs, Gαq, Gαi, and two Gβ subunits. 6) Many enzymes in the signaling cascade including adenylyl cyclase, PDE, PKA, PLC, PKC, and serine-threonine protein kinases. No GRs or ENaCs were expressed in any of the SCTs.

## Discussion

The gene expression profiles of single chemosensory neurons are described here for the first time in any crustacean, indeed in most animals except model organisms such as nematodes, fruit flies, mosquitoes, and mice. Our analysis is based on 14 transcriptomes from olfactory sensory neurons (OSNs) of the Caribbean spiny lobster, *Panulirus argus*, with half of the transcriptomes from single OSNs (single cell transcriptomes, SCT1a to SCT1g) and the other half from preparations containing two to 400 OSNs (multicell transcriptomes, MCT2 to MCT400).

### IR25a and IR93a as obligate co-IRs in OSNs of *P. argus*

Each SCT and MCT contained both IR25a and IR93a. This is consistent with previous immunocytochemical and in situ hybridization studies of *P. argus* showing that IR25a is expressed in all or virtually all OSNs [6, 7, 16, 48], as is IR93a [16]. In fact, IR25a also appears to be expressed in most if not all CSNs in the antennules, second antennae, and legs of *P. argus* [6, 7]. Thus, IR25a appears to be an obligate co-IR for all types of olfactory and chemosensory neurons in *P. argus*, but whether this is also true for IR93a requires more research. The number of transcripts per million in OSNs of *P. argus* appears to be about two times higher for IR93a than IR25a, suggesting that IR93a might be more highly represented in the heterotetrameric IRs than is IR25a.

Are IR25a and IR93a also obligate co-IRs in other crustacean species? Immunocytochemical studies of *H. americanus* indicate that IR25a is expressed in most or all OSNs [7, 47–49] although not in all CSNs of *H. americanus* [7]. In crustaceans more generally, IR25a is reported in copepods, branchiopods, amphipods, and decapods, but little information is available on which cells express it [6, 7, 16–24, 48, 50]. IR93a has been reported in the antennules and other chemosensory organs of all four species of decapod crustaceans in which it was examined, and in much higher abundance in the LF than dactyl [7]. This suggests that IR25a and IR93a are obligate co-IRs in OSNs and CSNs in many crustacean species, though more research is necessary to test this idea.

### Co-IRs IR8a and IR76b are not expressed in OSNs of *P. argus*

The co-receptors IR8a and IR76b are abundantly expressed in LF and dactyl of *P. argus* and three other decapod crustaceans. In *P. argus*, while IR8a has similar abundance in LF and dactyl, IR76b has higher abundance in dactyl than LF [6, 7]. Our study did not find either co-IRs in OSNs. An in situ hybridization study also failed to find IR8a expression in OSNs [16]. We hypothesize that IR8a and IR76b are expressed in CSNs and/or mechanosensory neurons in the bimodal sensilla of LF and not in OSNs. In *Drosophila*, IR25a, IR8a, and IR76b also have different expression patterns. IR8a expression is limited to antennae of adult flies while IR25a is expressed not only in adult antennae but also in other adult organs and in larvae [25]. IR76b is expressed in all sensory organs of flies that express IR25a [25] including antenna. Although the functions of IR8a or IR76b are unknown in *P. argus*, they are known for some insects. For example, the combined expression of IR8a and specific tuning IRs determines the odorant response specificity in OSNs of fruit flies and mosquitoes [43–46]. When expressed by itself with no tuning IRs, IR76b is sufficient for tasting salt [78, 113]. When co-expressed with IR41a, IR76b mediates detection of amines, whereas when co-expressed with IR20a, it mediates detection of amino acids [31]. When co-expressed with IR25a and IR56d, IR76b mediates responses to carbonation [25]. The roles of IR8a and IR76b in crustaceans are not yet known, but they appear to function in CSNs rather than OSNs.

### A diversity of tuning IRs is expressed in OSNs of *P. argus*

The total number of OSNs sampled in our 14 transcriptomes is ca. 500, which is low compared to the ca. 300,000 OSNs in a single LF of *P. argus* [5]. Even so, these 14 transcriptomes contain transcripts of 361 of the 578 identified tuning IRs in *P. argus*. This supports the idea that an aesthetasc with its ca. 320 OSNs represents the functional unit of olfaction in *P. argus* [114] and probably also in other crustacean species [115].

The specific combination of tuning IRs and co-IRs expressed in an OSN of *P. argus* is expected to determine its response specificity, as shown for flies and mosquitoes (e.g. [10, 27, 32, 41, 44–46]. Our results show that single OSNs of *P. argus* express 9 to 53 of the 578 tuning IRs, though only a few of them are expressed at high transcript levels. In five of the seven SCTs, one to three tuning IRs collectively accounted for over 95% of the total number of transcripts of tuning IRs, while in the other two SCTs, 6 to 8 tuning IRs were required to account for this level of expression. The identity of the highly expressed tuning IRs differed across the OSNs, though most are species-specific IRs and all have higher expression levels in LF than dactyl. Interestingly, three tuning IRs were expressed in every OSN, though at relatively low levels. These results suggest two features of the heterotetrameric receptor-channels formed by these IR subunits. First, a small number of coIRs and tuning IRs form most of the heterotetrameric IRs in a given OSN and thus the number of dominant heterotetramers may be relatively small per OSN. Second, a large number of minor heterotetramers may be possible, assuming that transcript levels reflect protein levels. This is consistent with a physiological study that concluded that single chemosensory neurons in the LF of *P. argus* can express more than one type of receptor molecule [115]. In *Drosophila*, the number of IRs expressed in single OSNs is two to five, including co-IRs [8]. Thus, spiny lobster OSNs appear to be generally similar to *Drosophil*a OSNs if considering only the most highly expressed tuning IRs, but considering the expression of the less abundant tuning IRs, the diversity of heterotetrameric IRs in spiny lobsters may be relatively greater.

The ratio of transcripts per million of co-IRs and tuning IRs is approximately the same across the OSNs (median ratio 1.0, range 0.5 to 1.5). The significance of this is not clear, though it raises the possibility that the typical heterotetrameric IR is composed of two co-IRs and two tuning IRs, as suggested by Abuin et al. [41] for *Drosophila* OSNs. More work is necessary to determine the combinations of co-IRs and tuning IRs that form the heterotetrameric IRs in O crustacean OSNs.

IRs in *Drosophila* and mosquitoes can contribute to the responsivity of chemosensory cells as well as thermo- or hygrosensory cells [37, 39, 43, 44, 116, 117]. For example, the combination of IR25a, IR93a, and IR40a in *Drosophila* specifies a humidity sensitive cell rather than a chemosensory cell [36, 37]. Interestingly, one OSN of *P. argus* (SCT1g) expressed the combination of IR25a, IR93a, and a member of the IR40a family (IR40a-3), and no other tuning IRs. The functional significance of such an expression pattern awaits future study.

### A plethora of TRP channels in *P. argus* OSNs, but what do they do?

Spiny lobster OSNs have a diversity of TRP channels, with representatives from both groups and most families though not TRPS [7, 57]. Members of the TRPA subfamily of spiny lobsters have higher abundance compared to other TRP subfamilies. Homologues of TRPA channels in *Drosophila* contribute to detecting chemicals (e.g. [118, 119]). For example, TRPA1, which has a homologue in all seven SCTs in spiny lobsters, is expressed in the labellum and mouthparts of *Drosophila* and detects aversive chemicals. Painless in *Drosophila* prevents male-male courtship and mediates avoidance of deterrent compounds. However, TRP homologues can also be involved in other senses than chemoreception, including photoreception, thermoreception, hygroreception, gravitoception, and proprioception [119]. Furthermore, the mechanisms underlying TRP channel activation in chemical senses can involve direct or indirect gating by chemical stimuli (e.g. [81, 95]. The functions of TRP channels in OSNs of spiny lobsters remain speculative, though there is physiological and pharmacological evidence for a channel with TRP-like properties that is involved in olfactory transduction in *P. argus* [92, 120, 121].

### GRs and ENaCs appear to be absent from OSNs in *P. argus*

GRs are an ancient lineage of chemoreceptors that have expanded families in insects and other arthropods [12, 67–69]. Although GRs have been identified in crustaceans, including in the antennules of decapod crustaceans, the number of different types of GRs per species is low, with the notable exceptions of *Daphnia, Hyalella azteca*, and *Eurytempora affinis* [6, 7, 16, 21–23, 70–72]. Only one GR was found in the antennules and legs of *P. argus* [6, 7], and it was not expressed in any of the SCTs. Rather, it was found only in the transcriptome containing 400 OSNs, and since this preparation likely contained cell types besides OSNs, it is not clear that the GR is expressed in chemosensory cells. Like GRs, ENaCs are involved in chemoreception in some clades including insects. While the LF and dactyls of *P. argus* have eight types of ENaCs [7], *P. argus* OSNs apparently lack them. Thus, it appears that GRs and ENaCs are not expressed in *P. argus* OSNs at all or at levels sufficient for them to play significant roles.

### NMDA receptors as potential modulators of OSNs

Of the five types of NMDArs found in the three organ-level transcriptomes of *P. argus* [6, 7], four types were identified in the SCTs of *P. argus*. Every SCT expressed at least one or two types of NMDArs. Especially abundant were homologues of NMDAr1 and NMDAr2d, though NMDAr2a and NMDAr2b were also present. These NMDArs may be targets of synaptic inputs from central neurons, akin to that described for the roles of histamine and GABA and their receptors in pre-synaptic inhibition in the olfactory lobes of *P. argus* [122–124]. No such modulatory effects have been shown in crustaceans for glutamate and NMDA receptors, but in *Drosophila* NMDAr1 might be expressed in at least one class of OSN and might be involved in activity-dependent remodeling of OSN-to-interneuron synapses in the antennal lobe [125]. Another possibility is that *P. argus* NMDArs could function non-synaptically by responding to circulating glutamate, which has been shown for other neurons [126].

### GPCRs and G proteins: modulation and candidate chemoreceptor genes?

GPCRs have diverse functions in sensory neurons across phyla, including as chemoreceptor proteins on dendrites of chemoreceptor cells [127, 128], as post-synaptic receptors on somata or axonal terminals that are modulated by central neurons [129–134], or as receptors sensitive to chemicals circulating in blood [129, 135]. Our finding that each OSN of *P. argus* expresses a dozen or more types of GPCRs including orphans whose ligands have not been characterized allows that some might function in *P. argus* OSNs as chemoreceptors of environmental chemicals.

Many of the GPCRs in the *P. argus* OSN transcriptomes are candidate receptors for neurotransmitters at synapses or for chemicals circulating in the hemolymph. These include homologues of receptors for amines including histamine, serotonin, dopamine, octopamine, and tyramine; metabotropic acetylcholine receptors; a purinergic (adenosine) receptor; receptors for neuropeptides including allatostatin and trissin; a prostaglandin receptor, and a HP1 receptor that may have innate immune functions against bacteria [136]. Interestingly, two of these receptors in OSNs of *P. argus* – 5HT2A and mAChR-B – are enriched in and potentially involved in regulating presynaptic terminals of OSNs of *Drosophila* as they do in other systems [134].

The five GPCRs that are expressed in every SCT are probably functioning as receptors for neurotransmitters or neuromodulators, though they might function as chemoreceptors. These include: a metabotropic histamine receptor; a dopamine/ecdysteroid receptor; CG13579, which is an orphan receptor in *Drosophila* with sequence similarity to the dopamine/ecdysteroid receptor; GPA2/GPB5_R2 receptor, which is a leucine-rich repeat-containing GPCR belonging to a subclass of glycoprotein hormone receptors; and an uncharacterized orphan receptor (Orphan5a).

GPCRs are well known as chemoreceptor proteins in the deuterostomes, including vertebrates (humans, mice, and other mammals [127, 128]) and echinoderms [88–90]. Although protostomes predominately use ionotropic receptors such as IR, ORs, and GRs in chemoreception, they sometimes use GPCRs. Examples include serpentine receptors in nematodes [96, 137–139], opsins in *Drosophila* [81], and other GPCR types in gastropods [83, 84], ticks [140], and insects [94, 95]. We found two opsins in our SCTs, MCTs, and LF transcriptome. One opsin was expressed in moderate levels in several of the SCTs and MCTs of *P. argus*. This opsin clustered distantly in phylogenetic trees with onychopsin sequences and was therefore classified as onychopsin-like (Rump, Kozma, and Derby unpublished data). Onychopsins are found in the Onychophora (velvet worms), which is a sister group to the Arthropoda [141–143]. While onychopsins have not been shown to have a chemosensory function in any animals, the high expression of an onychopsin-like gene in OSNs of *P. argus* suggests that it might function as a chemoreceptor in these cells. Alternatively, since opsins mediate thermosensation and hearing in *Drosophila* [144–147], the *P. argus* onychopsin-like gene might be a part of senses other than chemoreception. Alternatively, onychopsin might make OSNs light sensitive, providing a circadian sensitivity as other opsins do in central neurons [148, 149]. Additionally, a trace amine-associated receptor (TAAR)-like sequence was highly expressed in half of the SCTs and MCTs, though this may not be a homolog of TAARs since to date TAARs have been found exclusively in vertebrates [150–152].

A histamine H1 receptor (H1R) in LF of four decapod species (Rump, Kozma, and Derby, unpublished data) and in all seven SCTs of *P. argus* is interesting since H1Rs typically mediate excitation and until now have been found only in deuterostomes [153, 154]. Indeed, the histamine binding pocket of the *P. argus* H1R has high sequence similarity to the binding pocket in deuterostome H1Rs [153]. Histamine excites OSNs of *P. argus* [155], and so *P. argus* H1Rs might encode dendritic chemoreceptor proteins. Histamine is also a modulator in olfactory lobes of *P. argus*: histaminergic interneurons regulate activity of OSNs by presynaptically inhibiting them, but via a channel different from H1R – a histamine-gated chloride channel [122–124, 156–160]. This histamine-gated inhibitory chloride channel has been characterized pharmacologically and physiologically as an inhibitory ionotropic receptor, but its sequence is unknown.

We also detected several GPCRs for which we could not identify homologues. Lacking homologues, these uncharacterized GPCRs remain candidate chemoreceptors in OSNs of *P. argus*, especially since several of them are expressed at high levels in many SCTs, including one (Orphan5a) that was expressed in every SCT.

Having transduction based on GPCRs requires having G proteins and downstream signaling molecules. There are many of these in the OSNs of *P. argus*. For example, four Gα subunits – Gαo, Gαs, Gαq, and Gαi – with Gαo and Gαs being most abundant, and Gβ subunits were found in every seven SCTs. Interestingly, the Gα:Gβ ratio in SCTs was close to 1 (median 0.84, range 0.51 to 1.29). All four Gα subunits have homologues in insect olfactory cells, with Gαs activating adenylyl cyclase, Gαi inhibiting adenylyl cyclase, Gαq activating PLC, and Gαo having less well characterized targets [94]. The Gα subunits are present more broadly in arthropod olfactory systems [140, 161]. Homologues of Gαs, Gαq, and Gβ occur in the olfactory transduction cascade of *H. americanus* [92, 162–165]. Signaling molecules downstream to G proteins are also present in *P. argus* OSNs, including adenylyl cyclases, phosphodiesterases, PKAs, PLCs, PKCs, and serine-threonine PKs. A PLC-β and G protein coupled receptor kinase are present in olfactory tissue of *H. americanus* [163, 166]. Thus, GPCRs and downstream signaling molecultes are important in crustacean OSNs, though their roles in reception and modulation are in need of more study.

### Multiple putative transduction cascades in single OSNs

Individual chemosensory neurons in many species express multiple types of receptors. For example, the terminal organ of *Drosophila* has taste cells each of which expresses combinations of variant IRs, GRs, and/or TRP channels, including all three in the same cell [167]. Single taste cells in adult *Drosophila* can express IR25a and an ENaC channel, Ppk23 [33]. Badsha et al. [118] propose that some taste cells of *Drosophila* express a combination of GRs, TRPC channels, and G-protein pathways. Pheromone-sensitive OSNs in *Manduca* express metabotropic receptors with TRP channels downstream [95], and homologues of TRPC channels are present in some vertebrate chemoreceptor cells [168]. Plant-derived bitter compounds can be detected by gustatory cells in *Drosophila* using GPCR and non-GPCR pathways like TRPA1 [81]. Our study in *P. argus* shows that single OSNs also express genes for a multitude of putative chemoreceptor proteins. Chief among these are variant IRs, of which one to several types are expressed at high levels in individual OSNs plus several to many at low levels. Given that these IRs assemble as heterotetramers, the possible combinations are large. Individual OSNs of *P. argus* also express five to seven TRP channels, many GPCRs including some that are candidate chemoreceptors, and several G proteins, protein kinases, and protein lipase. Together with the physiological, biochemical, and molecular evidence supporting the idea that individual spiny lobster OSNs have two G protein-activated second messenger pathways that mediate excitatory and inhibitory responses, one using the second messenger cAMP and the other inositol phosphate [16, 91–93], it appears that the peripheral chemosensory neurons can be quite complex in their encoding abilities. Models have been proposed whereby ionotropic receptors and G-protein cascades interact in chemosensory transduction [16, 42, 52, 95], but if and how that happens in OSNs of *P. argus* remains largely speculative and should be a focus of future work. Finally, the high number and diversity of receptor molecules and presence of both excitatory and inhibitory transduction cascades in single OSNs of *P. argus* suggest that each cell carries a highly integrated message to the olfactory centers of the brain. Future work combining physiological recordings and transcriptomics of single OSNs could address the functional consequences of this molecular complexity.

## Conclusions

Our study demonstrates that the single cell transcriptomics approach can be applied to crustacean chemoreception. In fact, to our knowledge, this is the first report of the application of single cell transcriptomics to crustacean chemoreception and only the second of any cells in crustaceans [169]. To understand olfactory coding in spiny lobsters, we need to apply this technique to a much larger set of OSNs. The aesthetasc sensillum appears to be a morphological and functional unit in the crustacean olfactory organ [114, 115]. But for *P. argus*, with its ca. 320 OSNs per aesthetasc [5], nearly 600 different IRs, many TRP channels and candidate GPCR chemoreceptors, and potentially dozens to hundreds of different physiological classes of sensory neurons [114, 170], we will need to sequence up to several hundred OSNs to have a solid understanding of the molecular diversity of OSNs. Furthermore, only by performing single cell transcriptomics on cells whose chemical sensitivities are physiological characterized and by experimentally regulating the expression of receptor proteins in cells will we be able to determine the function of receptor proteins in crustacean chemosensory neurons.

## Methods

### Animals

One juvenile Caribbean spiny lobster (*Panulirus argus*) of 50 mm carapace length was used in this experiment. It was collected in the Florida Keys and transported to The Whitney Laboratory of the University of Florida where it was maintained communally in an aquarium with running seawater at 20–23 °C and fed a diet of shrimp. It was collected and retained under a Special Activity License issued by the Florida Fish and Wildlife Conservation Commission of the Division of Marine Fisheries Management. Formal approval from the Institutional Animal Care and Use Committee of Georgia State University or other ethics committees was not required since our work did not involve vertebrate animals. Nonetheless, our protocols complied with standard practices including collecting tissue and sacrificing animals using cold anesthesia.

### RNA isolation from individual odorant-sensitive OSNs

Olfactory sensory neurons demonstrated to be responsive to odorants were collected for RNA sequencing using an in situ preparation for imaging calcium responses and established methods [110, 171, 172]. In this preparation, a single annulus of the lateral flagellum of the antennule was excised and the cuticle on the side opposite the aesthetasc sensilla was removed to access the OSN somata. These annuli were enzymatically treated (10 min in ∼1 mg/ml trypsin, papain, collagenase in *Panulirus* saline (PS) containing the following (in mM): 486 NaCl, 5 KCl, 13.6 CaCl_2_, 9.8 MgCl_2_, and 10 HEPES, pH 7.9 with NaOH) and then mechanically cleaned.

Odorant-responsive OSNs were identified using calcium imaging. These preparations were placed in a microcentrifuge tube in PS containing the fluorescent calcium indicator (10 μM Fluo-4 AM) prepared with 0.06% Pluronic F-127 (Invitrogen, ThermoFisher Scientific, USA). The tube was shaken for 30–60 min on an orbital shaker (∼70 rpm). These preparations were then transferred into fresh PS and mounted on a 35 mm plastic Petri dish and placed on the stage of an inverted microscope (Olympus IX-71) equipped with a cooled CCD camera (ORCA R2, Hamamatsu, Japan) under the control of Imaging Workbench 6 software (INDEC Systems, USA). A standard FITC filter set (excitation at 510 nm, emission at 530 nm) was used for single-wavelength measurements. The somata region of the preparation was superfused with PS. The aesthetasc region of the preparation was provided with a separate perfusion flow of PS to deliver odorants exclusively to them. The principal odorant tested was an aqueous extract of TetraMarine, a commercially available marine fish food (Tetra, Spectrum Brands Pet, LLC, Blacksburg, Virginia, USA). Flakes of TetraMarine were powdered, dissolved in water, filtered through a 0.2 μm syringe filter, and diluted to a final test concentration of 500 mg/l. DL-Glutamate (500 mM) was also tested on some preparations. The odorant stream was switched with the flow of PS that otherwise continuously superfused the sensilla (both ∼250 μl/min) using a multichannel rapid solution changer (RSC-160, Bio-Logic, France). Chemical stimuli were released in 500-ms pulses, such that the concentrations of the stimuli at the cell did not exceed 500 μg/l for TetraMarine and 500 μM for DL-glutamate.

OSNs were collected using a fine suction pipette containing the following (in mM): 210 NaCl, 696 D-glucose, 10 HEPES, 0.1 CaCl_2_, 1 EGTA, pH 7.8; free calcium concentration ~ 10 nM. The pipette tip was transferred to a 0.2 ml DNase/RNase free PCR tube with 3 μl lysis buffer (LB) containing 2.5 mM dNTP, 1 U/μl RNAse inhibitor, and 0.1% Triton X-100 in nuclease-free water and prepared according to BGI protocol. The tube was then centrifuged at 10,000 rpm at 4 °C for 30 s and immediately stored on dry ice or at − 80 °C until sequenced.

Our focus was on single OSNs, but we also collected some samples containing more than one OSN. Of a total of 19 samples collected, 14 samples with RNA concentration > 0.26 ng/μl passed quality control for RNA sequencing. Of these 14 quality samples, 7 were from single OSNs, and we term these single cell transcriptomes (SCT1a, SCT1b, SCT1c, SCT 1d, SCT1e, SCT1f, SCT1g). Seven of the 14 transcriptomes were from 2 to ~ 400 OSNs: one was from 2 OSNs, one was from 3 OSNs, one was from 4 OSNs, three were from ~ 15 OSNs that were a clump of cells from a single aesthetasc and one was from ~ 400 OSNs from two adjacent aesthetasc cell clusters, and we called these multi-cell transcriptomes MCT2, MCT3, MCT5, MCT15a, MCT15b, MCT15c, and MCT400, respectively.

### Sequencing, de novo assembly, and transcript abundance estimation

#### Sequencing

Samples were shipped on dry ice to BGI (Cambridge, Massachusetts, USA) for quality assessment and sequencing of RNA using the BGISEQ-500 sequencing platform and smart-seq2 amplification and library construction. The read length was 100 paired end base pair reads. The number of reads per sample was > 40 million. After sequencing, raw reads were filtered, and adapter sequences, low quality reads, and contamination were removed prior to delivery.

#### De novo assembled transcriptome

A single reference transcriptome that was previously generated for *P. argus* [7] using the EvidentialGene (EVG) pipeline (https://f1000research.com/posters/5-1695) with raw reads sequenced from lateral flagella, walking leg dactyls, and brain of *P. argus* was used in our analyses. The de novo assembly of the transcriptome is described in detail in [7]. In brief, eight individual assemblies were generated using the de novo assemblers Trinity v.4.0, Trans-Abyss v1.5.3, Velvet v1.2.10, and OASES v0.2.09, and these assemblies were input to the EVG pipeline to generate a single refined transcriptome. TransDecoder (http://transdecoder.github.io/) was used to translate proteins, and CD-Hit [173] was used to remove redundancy. Analyses were performed on cd90 datasets following redundancy removal. Kraken2 v2.0.8-beta (https://ccb.jhu.edu/software/kraken2/) [174, 175] was used to identify bacterial, archaea, and viral sequences in the *P. argus* reference transcriptome. Kraken2 was used with default settings against NCBI’s RefSeq database for bacteria, archaea, and viruses.

Raw reads from 14 OSN transcriptomes were mapped to the previously annotated *P. argus* reference transcriptome and the abundance of transcripts in each transcriptome was estimated using RSEM v1.3.3 [112]. Transcripts per million (TPM) from this estimation were used for analyses here. DESeq2 [176] was used to compare expression between LF and brain tissues and between LF and dactyl tissues as described in [7] (Additional files 8 and 9).

### IR and iGluRs

Variant IR and iGluR sequences that are expressed in the lateral flagella of *P. argus* and that contain both Pfam domain regions that identify this receptor type (i.e., PF00063, which contains the M1, P, M2, S2, and M3 regions, and PF10613, which contains the S1 region) [177] were previously identified using InterProScan 5 (v5.28–67.0) [178], annotated, and published [6, 7]. Other sequences that had only one of the two Pfam domain regions of the variant IRs were previously identified using InterProScan and published, but unnamed [6, 7]. Some of these sequences with only one of the Pfam domain regions were found in abundance in our SCTs and MCTs. We generated additional evidence that they are Variant IRs by including them in a maximum likelihood tree with Variant IRs and ionotropic glutamate receptors (iGluRs) having both domains. Sequences were aligned using MAFFT [179, 180] and manually trimmed on Jalview [181, 182] to remove gaps. Tree was the built using IQ-Tree using ModelFinder, and confidence values were generated with ultrafast bootstrap (UFBoot) [183–186]. We found that these one-domain sequences clustered closely with the two-domain Variant IRs and not the iGluRs, providing support that they are indeed Variant IRs. Consequently, we included them in our analysis. Newly identified IRs with only one domain region were annotated following the naming system used in [7] with the suffixes ‘x’ or ‘y’ denoting that these sequences have a missing domain region. A double gradient heatmap for Fig. 3 was created using GraphPad Prism version 8.4.2 for Windows (GraphPad Software, San Diego, California USA).

### TRP channels, GRs, and ENaCs

Similar to IRs and iGluRs, TRP channels, GRs, and ENaCs were identified in our transcriptomes based on their reads mapped to the previously annotated *P. argus* reference transcriptome [7].

### GPCRs, G proteins, other signaling molecules

GPCRs, G proteins, and transduction enzymes in the *P. argus* reference transcriptome [7] were identified using InterProScan (for classification of protein families), TMHMM (for prediction of transmembrane helices in proteins), and then phylogenetically classified (Rump, Kozma, and Derby unpublished data) prior to mapping reads from our transcriptomes.

## Supplementary information

**Additional file 2.** Gene expression for variant IRs. Co-IRs are listed first. Tuning IRs are ordered in the following sequence: 1) IRs having at least one IR with > 100 TPM in a SCT; 2) IRs having at least one IR with > 100 TPM in a MCT; 3) IRs having 1 to 100 TPM in at least one SCT; 4) IRs having 1 to 100 TPM in at least one MCT; 5) IRs not expressed in any SCT but in at least one MCT; 6) IRs not expressed in any SCT or MCT. Yellow color indicates IR expression > 100 TPM. Blue color indicates tuning IRs expressed in all SCTs or all MCTs. Red color indicates IRs for which there are no transcripts in any SCTs or MCTs.

**Additional file 3.** Gene expression for TRP channels. Blue color indicates TRP channels expressed in all SCTs or all MCTs. Red color indicates TRP channels with no transcripts in all SCTs (top) or all SCT and MCTs (bottom).

**Additional file 4.** Gene expression for ENaCs. Red color indicates ENaCs with no transcripts in all SCTs or all MCTs.

**Additional file 5.** Gene expression for NMDA receptors. Blue color indicates NMDArs expressed in all SCTs or all MCTs. Red color indicates NMDArs with no transcripts in all SCTs or all MCTs.

**Additional file 6.** Gene expression for class A GPCRs. Yellow color indicates GPCR expression > 50 TPM. Blue color indicates tuning GPCRs expressed in all SCTs or all MCTs. Red color indicates GPCRs with no transcripts in all SCTs or all MCTs.

**Additional file 7.** Gene expression for G proteins and other molecules in signaling cascades. Blue color indicates tuning sequences expressed in all SCTs or all MCTs. Red color indicates sequences with no transcripts in all SCTs or all MCTs.

**Additional file 8.** DESeq2 comparison for LF vs. brain.

**Additional file 9.** DESeq2 comparison for LF vs. leg dactyl.

**Additional file 10.** Sequences for GPCRs, G-proteins, and others in signaling cascades.

## Data Availability

The datasets supporting the conclusions of this article are available in NCBI under BioProject accession PRJNA596786 [7]. Predicted protein sequences for GPCRs, G-proteins, and other molecules in signaling cascades are available in Additional file 10.txt.

## Abbreviations

ENaC: Epithelial sodium channel
GPCR: G protein coupled receptor
GR: Gustatory Receptor
IR: Variant Ionotropic Receptor
LF: Lateral flagella of the antennules
MCT: Multi-cell transcriptome
NMDAr: *N*-methyl-D-aspartate receptor
OR: Olfactory receptor
OSN: Olfactory sensory neuron
SCT: Single cell transcriptome
TRP channel: Transient receptor potential channel
